# Light Emitting Diode Fluorescence Microscopy increased the detection of smear-positives during follow-up of Tuberculosis patients in India: program implications

**DOI:** 10.1186/s13104-015-1584-z

**Published:** 2015-10-23

**Authors:** Badri Thapa, Lord Wasim Reza, Ajay MV Kumar, Ashish Pandey, Srinath Satyanarayana, Sarabjit Chadha

**Affiliations:** Tuberculosis Department, International Union Against Tuberculosis and Lung Disease, South-East Asia Regional Office, C-6, Qutub Institutional Area, 110016 New Delhi, India

**Keywords:** LED-fluorescence microscopy, Follow-up smears, TB patients

## Abstract

**Background:**

In India, since July 2012, at designated Microscopy Centers (DMCs) in 200 medical colleges, sputum smear examination for tuberculosis bacilli changed from Ziehl Neelsen (ZN) method to auramine based Light Emitting Diode Fluorescent Microscopy (LED-FM) method. We assessed the additional yield of smear positives among patients undergoing follow-up sputum examination during TB treatment before and after deploying LED-FM.

**Methods:**

This was a before and after comparison study in eight conveniently selected medical college DMCs across North India. We extracted data from TB laboratory registers on number of TB patients examined for follow-up and their smear microscopy results including the grades by ZN (before; July–December 2011) and LED-FM (after; July–December 2012) and compared them.

**Results:**

Altogether, 2868 TB patients were examined by LED-FM and 2740 were examined by ZN during follow-up. LED-FM increased the proportion of follow-up smear positives from 5.0 % (n = 136) to 7.4 % (n = 213) with an additional yield of 77 follow-up smear-positives—with the highest increase in smears graded scanty (2.6 vs 1.2 %) (*p* value <0.05).

**Conclusions:**

Since all smear positives during follow-up are considered ‘presumptive multidrug resistant (MDR)-TB patients’ in India, introduction of LED-FM would result in additional number of patients eligible for MDR-TB testing, which would have otherwise been missed by ZN.

## Background

The World Health Organization (WHO) recommends phasing-in Light Emitting Diode Fluorescent Microscopy (LED-FM) as an alternative to conventional Ziehl-Neelsen microscopy (ZN) for diagnosis of smear-positive pulmonary tuberculosis (TB) [1]. Many studies in controlled laboratory settings have shown that LED-FM is about 10 % more sensitive and as specific as ZN in detecting TB bacillus on smear microscopy [2]. In India, since July 2012 under the Revised National Tuberculosis Control Programme, the sputum smear examination for TB bacilli at designated microscopy centers (DMCs) in 200 medical colleges, changed from ZN method to auramine based LED-FM method. LED-FM has been shown to increase the detection of smear positive tuberculosis under routine programmatic conditions [3].

Unlike at the time of diagnosis, TB patients who undergo follow-up sputum smear examination during TB treatment, produce less sputum and are mostly pauci-bacillary [4]. There is no evidence from India if use of LED-FM increases the detection of smear positives among patients undergoing follow-up sputum smear examination under routine programmatic conditions. In this study, we evaluated whether implementation of LED-FM increased the yield of smear positives during follow-up and discuss its programmatic implications.

## Methodology

We conducted a before and after comparison study in eight conveniently selected medical colleges in North India that were implementing LED-FM services. The study sample included all TB patients who underwent follow-up sputum smear examination in these colleges during the period July–December 2012 (using LED-FM) and July–December 2011 (using ZN microscopy). TB patients, as a part of monitoring their response to treatment, undergo follow-up smear examination at the end of intensive phase, 2 months into continuation phase and at the end of treatment. Those found to be sputum smear positive are evaluated for drug resistance. Patient details, reasons for sputum smear examination (whether for diagnosis or for follow-up) and results are recorded in the TB laboratory register. For this study, we included all patients who under-went follow-up sputum smear examination conducted during the two study periods. We reviewed the TB laboratory registers and abstracted aggregate data on number of TB patients examined for follow-up and their smear microscopy results including grades.

LED-FM and ZN procedures were performed by trained laboratory technicians. As per programme guidelines, smears were prepared from direct sputum specimens in both techniques. Briefly, for LED-FM, the heat fixed smears were flooded with 0.1 % auramine phenol for 7–10 min, rinsed with running water, decolorized with 3 % acid alcohol for 2 min twice, rinsed with running water, counter stained with 0.1 % potassium permanganate for 30 s, rinsed with running water and air dried [4]. For ZN microscopy, the heat fixed smears were flooded with 1 % carbol fuchsin for 5 min, rinsed gently with running water, decolorized with 25 % sulphuric acid for 2–4 min, rinsed with water, counter stained with 0.1 % methylene blue for 30 s, rinsed with running water and air dried [5]. Trained laboratory technicians prepared and examined the smears and reported the results as shown in Table 1 [3]. The microscopy centers performed smear microscopy procedures under a system of external quality assurance which included onsite visits by dedicated laboratory supervisors and periodic, random blinded rechecking of smears [6, 7]. As per national guidelines, two smears are required to be examined for follow up examinations [6]. Patient will be labelled as smear positive at follow-up if at least one smear is positive irrespective of the grades. If both smears are positive, the higher microscopy grade is used for reporting. As per the newer national guidelines on programmatic management of drug resistant TB, all TB patients who are smear positive during follow-up at the end of 2 months of intensive phase and/or 5 months of continuous phase (depending upon the districts) are considered as ‘presumptive multidrug resistant (MDR)-TB patients’ and are investigated for Rifampicin and Isoniazid resistance using culture and drug susceptibility test (CDST) [8]. If patients are confirmed to be having strains that are resistant to Rifampicin in vitro, they are treated using WHO-recommended standardized second line regimen.

**Table 1:** Guidelines of reporting sputum smear microscopy adopted by National Tuberculosis Control Programme in India

IUATLD/WHO scale (1000 × field = HPF*) Grades	Ziehl Neelsen (ZN) microscopy (1000 × magnification: 1 length = 2 cm = 100 HPF)	LED-Fluorescence microscopy (400 × magnification: 1 length = 40 fields = 200 HPF)
Negative	Zero AFB/1 length	Zero AFB/1 length
Positive		
Scanty	1–9 AFB/1 length or 100 HPF	1–19 AFB/1 length or 100 HPF
1+ grade	10–99 AFB/1 length or 100 HPF	20–199 AFB/1 length
2+ grade	1–10 AFB/1 HPF on average	5–50 AFB/1 HPF on average
3+ grade	>10 AFB/1 HPF on average	>50 AFB/1 HPF on average

* *HPF* high power field

The number of TB patients examined during follow-up and proportion found smear positive was computed, separately for LED-FM and ZN microscopy. Chi squared test was used to test statistical significance (p-value ≤0.05) (SPSS v16). Ethics approval for this study was obtained from the Ethics Advisory Group of International Union Against Tuberculosis and Lung Disease (The Union).

## Results

Of 2868 TB patients examined by LED-FM during follow-up, 213 (7.4 %) were smear-positive and of 2740 examined by ZN, 136 (5.0 %) were smear-positive—an additional yield of 77 follow-up smear-positives. The disaggregated results of first and second specimens are shown in Table 2. The additional yield of second specimen (negative on first specimen and positive only on second specimen) was 12.5 % with ZN microscopy whereas it was 21.6 % with LED-FM (p = 0.3).

**Table 2:** Performance of LED-FM (July–December 2012) in comparison to ZN microscopy (July–December 2011) in detecting AFB during follow-up of TB patients in eight medical colleges of India

Technique	Number examined	Number smear positive	PP	PN	NP	Proportion smear positive^#^	Additional Yield of second specimen (NP/all positives)^
ZN	2740	136	110	9	17	5.0 %	12.5 %
LED-FM	2868	213	149	18	46	7.4 %	21.6 %
Absolute increase (relative increase)	+128 (4.7 %)	+77 (56.6 %)				+2.4 % (48 %)	

*ZN* Ziehl Neelsen Microscopy, *LED-FM* light emitting diode fluorescence microscopy, *AFB* acid fast bacilli, *PP* first and second smear positive, *PN* first smear positive and second smear negative, *NP* first smear negative and second smear positive

^#^p value < 0.001

^ p value = 0.03

Compared to the before period, in the after-period there was a 5 % increase in the number of TB patients who underwent follow-up sputum smear examination. However, the number of TB patients found to be sputum smear positive rose by more than 50 % (Table 2). Applying 5.0 % positivity (observed in 2011) for the additional number of patients examined in 2012, LED-FM services still resulted in an additional yield of 71 follow-up smear-positives. Most of the increase was noted in smears graded scanty followed by 3+ (p-value < 0.05) (Table 3). The direct yield was higher by LED-FM in most (n = 7) sites compared.

**Table 3:** Comparison between Ziehl Neelson (ZN) and Light Emitting Diode-Fluorescence Microscopic technique (LED-FM) in detecting AFB (by smear quantification) during follow-up of TB patients in eight medical colleges of India

Smear grade of patient		ZN Microscopy 2011		LED-FM 2012		Change in percentage (95 % CI)	p-value
		n1	%	n2	%		
Negative		2604	95.0	2655	92.6	−2.4 (−1.1 to −3.7)	<0.0001
Positive	Scanty	33	1.2	75	2.6	1.4 (0.7–2.0)	0.0001
	1+	57	2.1	78	2.7	0.6 (−0.1 to 1.4)	0.14
	2+	29	1.1	27	0.9	−0. 2 (−0. 7 to 0. 4)	0.45
	3+	17	0.6	33	1.1	0.5 (0.0 to 1.0)	0.01
Total		2740	100.0	2868	100.0	–	–

*ZN* Ziehl Neelsen Microscopy, *LED-FM* light emitting diode fluorescence microscopy, *AFB* acid fast bacilli

## Discussion

This is the first study from India examining the impact of LED-FM on the sputum-positivity during follow-up of TB patients under routine programmatic settings. The increase in yield by LED-FM could be due to (1) increased absorbability of mycolic acid and damaged bacilli to auramine than fuschin, (2) the LED-FM stained background of slide is brighter, making it easier for laboratory technicians to focus and examine slides with fewer bacilli and (3) increased area of the slide examined [7, 9]. Since these findings come from a setting of quality-assured smear microscopy under routine programmatic settings, they are most likely to be accurate and reflect operational realities and importantly during the period of study external quality assurance did not yield any major errors.

These results have important programme implications. LED-FM leads to an increase in the number of TB patients who will be identified as “presumptive MDR-TB patients” who will require CDST with consequent increase in laboratory workload. Several studies in the past have shown that nearly two-thirds of the smear positives during follow-up were culture negative, especially so if the smears are graded scanty, which most likely represent dead, non-viable bacilli, yet to be excreted out of the body [10, 11]. Hence, it is not clear if increase in identification of follow-up smear-positives using LED-FM is actually beneficial in enhancing detection of MDR-TB cases or just unnecessary additional workload for the CDST laboratories. Since we do not have information on culture results in our study, the possibility of false positive results could not be assessed and should be a topic for future studies. To avoid this ‘smear-positive culture-negative’ phenomenon and increase the specificity of smear microscopy during follow-up, The Union in their TB guidelines recommend that all follow-up smear-positives (especially that done during month 5) in the first sample be reconfirmed by a second positive smear using an early morning sputum sample before declaring as ‘treatment failure’ and start next steps including investigation for MDR-TB [11]. A recent study from Chennai showed that if only one of the two smears was positive, this is most likely to be scanty and hence culture negative [12]. Adopting the Union’s approach would mean that TB patients who are positive on both the smears only would be investigated for MDR-TB and would thus reduce unnecessary workload. The other alternatives to reduce unnecessary cultures are to use new methods like vital staining with fluorescein diacetate or slide culture, but these are not widely available or validated under routine conditions [13–15].

There were a few limitations to this study. It would have been better if the same samples were processed with the two methods. However, this could not be performed as the ZN microscopy system was already replaced with the LED-FM microscopy in the study sites. The culture and DST results were unavailable and hence it could not be found out if use of LED-FM actually increased the detection of MDR-TB cases. As in all pre-post designs, it is difficult to attribute the entire change to the intervention (LED-FM) as there could be other undocumented changes (for example, pre-treatment smear positivity) during the study period. While we do not suspect this, we have no data to rule it out completely and is one of the limitations. Also the study did not disaggregate the follow-up smears at end of intensive phase and end of treatment. Since the study was conducted in conveniently selected medical colleges, there may be difficulties in generalizing the results to whole of India. Further studies should collect data countrywide or from a representative sample to confirm these results and act upon them.

## Conclusions

Implementation of LED-FM led to increase in identification of follow-up smear-positives among TB patients, mostly graded scanty. This has important resource implications for the TB programme in India.

## References

[1] World Health Organization (2010). Fluorescent light emitting diode (LED) microscopy for diagnosis of tuberculosis: policy statement.

[2] Steingart KR, Henry M, Ng V, Hopewell PC, Ramsay A, Cunningham J (2006). Fluorescence versus conventional sputum smear microscopy for tuberculosis: a systematic review. Lancet Infect Dis.

[3] Reza LW, Satyanarayana S, Pandey A, Kumar S, Pandey A, Devendrappa NM (2013). LED fluorescence microscopy increases the detection of smear-positive pulmonary tuberculosis in medical colleges of India. Public Health Action.

[4] Nataraj G, Kanade S, Parikh R, Khatri V, Mehta P, Athavale A (2011). Incremental yield in sputum smear positivity by examining a second early morning sputum specimen in follow-up patients on DOTS: 7 year analysis of RNTCP laboratory register. Indian J Tuberc.

[5] Central Tuberculosis Division: Module for laboratory technician. New Delhi: Directorate General of Health Services, Ministry of Health and Family Welfare, Government of India; 2005.

[6] Central Tuberculosis Division: Manual for sputum smear fluorescence microscopy. New Delhi: Directorate General of Health Services, Ministry of Health and Family Welfare, Government of India; 2011.

[7] Central Tuberculosis Division: Revised National Tuberculosis Control Program Technical and operational guidelines for tuberculosis control. New Delhi: Directorate General of Health Services, Ministry of Health and Family Welfare, Government of India; 2005.

[8] Xia H, Song YY, Zhao B, Kam KM, O’Brien RJ, Zhang ZY (2013). Multicentre evaluation of Ziehl-Neelsen and light-emitting diode fluorescence microscopy in China. Int J Tuberc Lung Dis.

[9] Central Tuberculosis Division: Guideline on programmatic management of drug resistant TB (PMDT) in India. Revised National Tuberculosis Control Program: Directorate General of Health Services, Ministry of Health and Family Welfare, Government of India; 2012.

[10] Reza LW, Satyanarayna S, Enarson DA, Kumar AM, Sagili K, Kumar S (2013). LED-fluorescence microscopy for diagnosis of pulmonary tuberculosis under programmatic conditions in India. PLoS One.

[11] Chamie G, Charlebois ED, Srikantiah P, Walusimbi-Nanteza M, Mugerwa RD, Mayania H (2010). *Mycobacterium tuberculosis* microbiologic and clinical treatment outcomes in a randomized trial of immediate versus CD4^+^-initiated antiretroviral therapy in HIV-infected adults with a high CD4^+^ cell count. Clin Infect Dis.

[12] Enarson DA, Reider HL, Arnadottir, Trebucq A. Management of tuberculosis. 5th ed. France: International Union Against Tuberculosis Control and Lung Disease; 2000.

[13] Kumar RS, Kumar AMV, Claassens M, Banurekha VV, Gomathi NS, Venkatesan P (2013). Number of sputum specimens during treatment follow-up of Tuberculosis patients—two or one?. Pub Health Act.

[14] Van Deun A, Maug AKJ, Hossain A, Gumusboga M, de Jong BC (2012). Fluorescein diacetate vital staining allows earlier diagnosis of rifampicin-resistant tuberculosis. Int J Tuberc Lung Dis.

[15] Hemavathi SP, Ramesh DH (2012). Comparative evaluation of the rapid slide culture and microscopy with the conventional culture method in the diagnosis of pulmonary tuberculosis. J Clin Diag Res.

